# The genus *Boletopsis* (Bankeraceae, Thelephorales) in China

**DOI:** 10.3897/BDJ.13.e142835

**Published:** 2025-05-09

**Authors:** Pei Lyu, Wei Zeng, Hu-Hua Yang, En-De Liu, Yan-Chun Li

**Affiliations:** 1 State Key Laboratory of Phytochemistry and Natural Medicines, Kunming, China State Key Laboratory of Phytochemistry and Natural Medicines Kunming China; 2 Yunnan Key Laboratory for Fungal Diversity and Green Development, Kunming, China Yunnan Key Laboratory for Fungal Diversity and Green Development Kunming China; 3 University of Chinese Academy of Sciences, Beijing, China University of Chinese Academy of Sciences Beijing China; 4 Southwest Survey and Planning Institute of the National Forestry and Grassland Administration, Kunming, China Southwest Survey and Planning Institute of the National Forestry and Grassland Administration Kunming China; 5 The Administration Bureau of Jinguang Temple Provincial Nature Reserve, Dali, China The Administration Bureau of Jinguang Temple Provincial Nature Reserve Dali China; 6 Key Laboratory for Plant Diversity and Biogeography of East Asia, Kunming Institute of Botany, Chinese Academy of Sciences, Kunming, China Key Laboratory for Plant Diversity and Biogeography of East Asia, Kunming Institute of Botany, Chinese Academy of Sciences Kunming China

**Keywords:** edible mushroom, morphology, molecular phylogeny, taxonomy, new species

## Abstract

**Background:**

*Boletopsis* is a genus of the family *Bankeraceae* with eight accepted species in the world. Species in the genus are ectomycorrhizal fungi and edible. In this study, we conducted a phylogenetic analysis of this genus, based on ITS and nrLSU sequences, using Maximum Likelihood and Bayesian Inference, along with morphological observations in the field and under microscopies. Three species of *Boletopsis* from China were described and illustrated, including two known species, viz., *B.macrocarpa* and *B.tibetana* and one new species, namely *B.longipes*. A global key to the genus is also provided.

**New information:**

The new species can be distinguished from other species of *Boletopsis* by its pastel brown pileus when mature, slender stipes measuring 5–13 × 2–3 cm, large basidia, measuring 15–36 × 7–15 μm, solitary or gregarious in mixed forests dominated by trees of the families *Pinaceae* and *Fagaceae* at a relatively low altitude about 1000–2600 m. Line drawings of microstructures, colour pictures of fresh basidiomata and detailed descriptions of the new and two known *Boletopsis* species from China are provided. In addition, a key to the accepted species of the genus worldwide is provided.

## Introduction

The genus *Boletopsis* Fayod was originally established, based on *Boletusleucomelas* Pers. [= *Boletopsisleucomelaena* (Pers.) Fayod] by Fayod from Europe (Fayod 1889). The features of *Boletopsis* are the annual terricolous basidiomata, poroid hymenophore, central to eccentric stipes, irregularly polygonal or angular to tubercular basidiospores and monomitic hyphal system with clamp connections (Niemelä and Saarenoksa 1989, Watling and Milne 2008, Cooper and Leonard 2012, Cooper and Leonard 2012, Zhou et al. 2022, Vizzini et al. 2023). It is an ectomycorrhizal fungal genus in the family *Bankeraceae* and associated with trees in the families *Pinaceae* and *Fagaceae* (Watling and Milne 2006, Watling and Milne 2008, Zhou et al. 2022). Nowadays, there are eight species universally accepted in the genus *Boletopsis*, including *B.grisea* (Peck) Bondartsev & Singer, *B.leucomelaena* (Pers.) Fayod, *B.macrocarpa* Y.C. Dai, F. Wu & H.M. Zhou, *B.mediterraneensis* G. Moreno, Carlavilla, Bellanger, Olariaga, P.-A. Moreau, Bidaud, Loizides & Manjón, *B.nothofagi* J.A. Cooper & P. Leonard, *B.smithii* K.A. Harrison, *B.tibetana* Y.C. Dai, F. Wu & H.M. Zhou and *B.watlingii* Blanco-Dios (Blanco-Dios 2018). For the other species once reported in *Boletopsis*: *B.subsquamosa* (L.) Kotl. & Pouzar was considered synonymous with *Albatrellusovinus* (Schaeff.) Kotl. & Pouzar (Donk 1974, Niemelä and Saarenoksa 1989, Ryvarden and Gilbertson 1993) and *B.subcitrina* Corner and *B.atrata* Ryvarden were transferred to the genus *Corneroporus* by Hattori and Vizzini et al., respectively (Hattori 2001, Vizzini et al. 2023). *Boletopsis.macrocarpa* and *B.tibetana* were originally described from East Asia (Zhou et al. 2022), while *B.grisea*, *B.leucomelaena*, *B.mediterraneensis* and *B.watlingii* were originally described from Europe (Niemelä and Saarenoksa 1989, Watling and Milne 2006, Crous et al. 2019). *Boletopsissmithii* was originally described from North America (Watling and Milne 2008) and *B.nothofagi* was originally described from New Zealand (Cooper and Leonard 2012).

In China, species of *Boletopsis* are edible mushrooms and sold in local markets as “black bear paw” (Zhou et al. 2022). In Japan, the *Boletopsis* species are edible mushrooms locally called “kurokawa” (Shimosato et al. 2013). So far, four species have been recognised from China, including two formally described species from southwest China, viz. *B.macrocarpa* distributed in pure *Pinus* forests at an altitude ranging from 2400 m to 3400 m (Zhou et al. 2022) and *B.tibetana* distributed in mixed forests dominated by trees of the genera *Picea* and *Quercus* or in pure *Picea* forests an altitude about 2900 m to 3350 m (Zhou et al. 2022). In addition, there are two potential new species recognised by Zhou et al. (2022) from China, but unclarified due to the paucity of materials. During our field investigation, the authors encountered a peculiar *Boletopsis* species from Laojun Mountain, a part of Hengduan Mountains, located in Yunnan Province, southwest China. Based on morphological and molecular phylogenetic studies, this species is different from the known species and the above two unclarified species. In this study, the new species and the two known species from China were illustrated and documented.

## Materials and methods

**Sampling and morphological study**

Samples were collected from southwest China and dried in an electric drier, then deposited in the fungal herbarium of the Herbarium Kunming Institute of Botany, Chinese Academy of Sciences (KUN-HKAS). Photographs and notes of fresh basidiomata were taken in the field. Colour codes were recorded, based on Kornerup & Wanscher (Kornerup and Wanscher 1981).

For morphological studies, materials were sectioned from dried samples and rehydrated in 10% potassium hydroxide (KOH). A Leica DM2000 light microscope was used to observe micro-morphological characteristics and ZEISS Sigma 300 scanning electron microscope (SEM) was used to observe basidiospore ornamentations. All line drawings were made under a microscope by freehand.

In the description of basidiospores, the notation [n/m/p] stands for n basidiospores measured from m basidiomata of p collections. The form (a)b–c(d) is used to describe the dimensions of basidiospores, in which range b–c contains at least 90% of the measured values, while a and d in parentheses represent extreme values. Q is the length/width ratio of a basidiospore; Q_m_ is the average Q of all basidiospores measured ± sample standard deviation.

**DNA extraction, amplification and sequencing**

Genomic DNA was extracted using 2× CTAB solution (Coolaber Technology Co., Ltd, Beijing, China) following the manufacturer’s instructions. The internal transcribed spacer (ITS) and the large subunit of nuclear ribosomal RNA (nrLSU) were amplified with primers ITS1/ITS4 or ITS5/ITS2 and 5.8SR/ITS4 and LROR/LR3 (White et al. 1990, Vilgalys Lab 1992), respectively. PCR reactions contained 1 μl DNA solution (adjusted to approximately 50 ng), 1 μl of each primer and 15 μl 2× Taq PCR Master Mix including Taq DNA Polymerase, MgCl2 and dNTP mix (Beijing Biomed Gene Technology Co., Ltd., Beijing, China). The final volume was adjusted to 50 μl with distilled sterile H_2_O. The amplification conditions were set as follows: denaturation at 95◦C for 4 min, 35 cycles of 30 s at 94◦C, 40 s at 50◦C, 1 min at 72◦C, a final extension of 8 min at 72◦C and then coolant at 14◦C. The PCR amplification products were sequenced using Sanger sequencing by Sangon Bioengineering (Shanghai) Co., Ltd.

**Phylogenetic analyses**

DNA sequences were compiled with SeqMan (DNASTAR Lasergene 9). Sixteen sequences (nine of ITS and seven of nrLSU) from nine collections were newly generated in this study and aligned with selected sequences downloaded from GenBank and previous studies (Watling and Milne 2008, Cooper and Leonard 2012, Crous et al. 2019, Zhou et al. 2022) (Table 1). *Sarcodonimbricatus* (L.) P. Karst. was selected as outgroup taxa. Sequences were aligned with MAFFT v.7.490 and adjusted manually with PhyDe if necessary.

The phylogenetic analyses of combined dataset (ITS + nrLSU) were conducted with Maximum Likelihood (ML) and Bayesian Inference (BI). The ML analysis was conducted in Raxml GUI2.0.10 under the default model GTR + G + I (Edler et al. 2021) with 1,000 bootstrap replicates. For BI analysis, the best-fit partition model: GTR + F + G4 for ITS, and GTR + F + I for nrLSU were selected in PhyloSuite v.1.2.3 (Zhang et al. 2020) with plug-in ModelFinder v.2.2.0 (Kalyaanamoorthy et al. 2017) and plug-in MrBayes v.3.2.7a (Ronquist et al. 2012) was used with 1,000,000 replicates with the average standard deviation of split frequencies 0.0039 (< 0.01).

## Taxon treatments

### Boletopsis longipes

Yan C. Li & P. Lyu sp. nov.

57D8D2DC-3902-5AAA-AD79-62E0E9B0DC46

MB856495

#### Materials

- **Type status:** Holotype. **Occurrence:** recordNumber: Yan-Chun Li 4984; recordedBy: Yan-Chun Li; individualID: KUN-HKAS 136927; occurrenceID: 29E115A4-0607-5974-AE41-A551CD8C5138; **Taxon:** scientificName: *Boletopsislongipes* Yan C. Li & P. Lyu; kingdom: Fungi; phylum: Basidiomycota; class: Agaricomycetes; order: Thelephorales; family: Bankeraceae; genus: Boletopsis; **Location:** higherGeography: East Asia; China; Yunnan; municipality: Lijiang; locality: Yulong Naxi Autonomous County, Laojun Mountain; verbatimElevation: 2559 m; verbatimLatitude: 26.8451°N; verbatimLongitude: 99.8461°E; **Event:** eventDate: 2023-08-2
- **Type status:** Paratype. **Occurrence:** recordNumber: Yan-Chun Li 5006; recordedBy: Yan-Chun Li; individualID: KUN-HKAS 136928; occurrenceID: BD33EB8B-2D6F-551A-AE9A-7C499E811D5A; **Taxon:** scientificName: *Boletopsislongipes*; kingdom: Fungi; phylum: Basidiomycota; class: Agaricomycetes; order: Thelephorales; family: Bankeraceae; genus: Boletopsis; taxonRank: species; **Location:** higherGeography: East Asia; China; Yunnan; municipality: Lijiang; locality: Yulong Naxi Autonomous County, Laojun Mountain; verbatimElevation: 2559 m; verbatimLatitude: 25.2524°N; verbatimLongitude: 99.3136°E; **Event:** eventDate: 2023-08-25
- **Type status:** Paratype. **Occurrence:** recordNumber: Mei-Xiang Li 37; individualID: KUN-HKAS 113275; occurrenceID: 3660E32E-7F39-5FA2-9E58-25733FE4D09D; **Taxon:** scientificName: *Boletopsislongipes*; kingdom: Fungi; phylum: Basidiomycota; class: Agaricomycetes; order: Thelephorales; family: Bankeraceae; genus: Boletopsis; **Location:** higherGeography: East Asia; China; Yunnan; municipality: Honghe; locality: Lvchun County, Huanglian Mountain; verbatimElevation: 1000-1600 m; **Event:** eventDate: 2020-07-30
- **Type status:** Paratype. **Occurrence:** recordNumber: Yan-Chun Li 1724; recordedBy: Yan-Chun Li; individualID: KUN-HKAS 59471; occurrenceID: DCB88158-507C-5A07-802A-DAF9A8A5A550; **Taxon:** scientificName: *Boletopsislongipes*; kingdom: Fungi; phylum: Basidiomycota; class: Agaricomycetes; order: Thelephora; family: Bankeraceae; genus: Boletopsis; **Location:** higherGeography: East Asia; China; Yunnan; county: Baoshan; locality: Longyang, Haitang Village; verbatimElevation: 2340 m; verbatimLatitude: 25.2524°N; verbatimLongitude: 99.3136°E; **Event:** eventDate: 2009-07-21
- **Type status:** Paratype. **Occurrence:** recordNumber: Yan-Chun Li 1735; recordedBy: Yan-Chun Li; individualID: KUN-HKAS 59482; occurrenceID: 7B7429C6-A999-56CF-B25E-35EB64E1EFF3; **Taxon:** scientificName: *Boletopsislongipes*; kingdom: Fungi; phylum: Basidiomycota; class: Agaricomycetes; order: Thelephorales; family: Bankeraceae; genus: Boletopsis; **Location:** higherGeography: East Asia; China; Yunnan; county: Baoshan; locality: Longyang, Haitang Village; verbatimElevation: 2340 m; verbatimLatitude: 25.2524°N; verbatimLongitude: 99.3136°E; **Event:** eventDate: 2009-07-22
- **Type status:** Paratype. **Occurrence:** recordNumber: Yan-Chun Li 5844; recordedBy: Yan-Chun Li; individualID: KUN-HKAS 146814; occurrenceID: 985083D5-5A44-5287-8806-2CC18AAC20AA; **Taxon:** scientificName: *Boletopsislongipes*; kingdom: Fungi; phylum: Basidiomycota; class: Agaricomycetes; order: Thelephorales; family: Bankeraceae; genus: Boletopsis; **Location:** higherGeography: East Asia; China; Yunnan; stateProvince: Dali Bai Autonomous Prefecture; county: Yongping; locality: Jinguang Temple Provincial Nature Reserve; verbatimElevation: 2371 m; verbatimLatitude: 25.1586°N; verbatimLongitude: 99.5161°E; **Event:** eventDate: 2024-07-27

#### Description

Basidiomata annual, medium to large, centrally stipitate, solitary to gregarious (Fig. 1a, b, c). Pileus 6.5–17 cm in diameter, convex to flat, slightly concave at centre when mature, black (4F8) to dark grey (13D1) when young, turning pastel brown (6D5–6E5) when mature; surface covered with concolorous to bark brown (3F3–3F4) tomentose squamules when mature or aged; margin undulate. Context 0.7–1.3 cm thick at centre, white (1A1), darkening when bruised, becoming light grey (28A2) when dry. Hymenophore slightly decurrent; surface white (1A1), becoming light grey (28A2) when damaged, pores subangular to angular or irregular, 2–4 per mm; tubes concolorous with hymenophoral surface, darkening when bruised, up to 0.3 cm long. Stipe 5–13 × 2–3 cm, solid, clavate to subcylindrical, concolorous with pileal surface or much paler; basal mycelium white (2A1).

**Figure 2.**
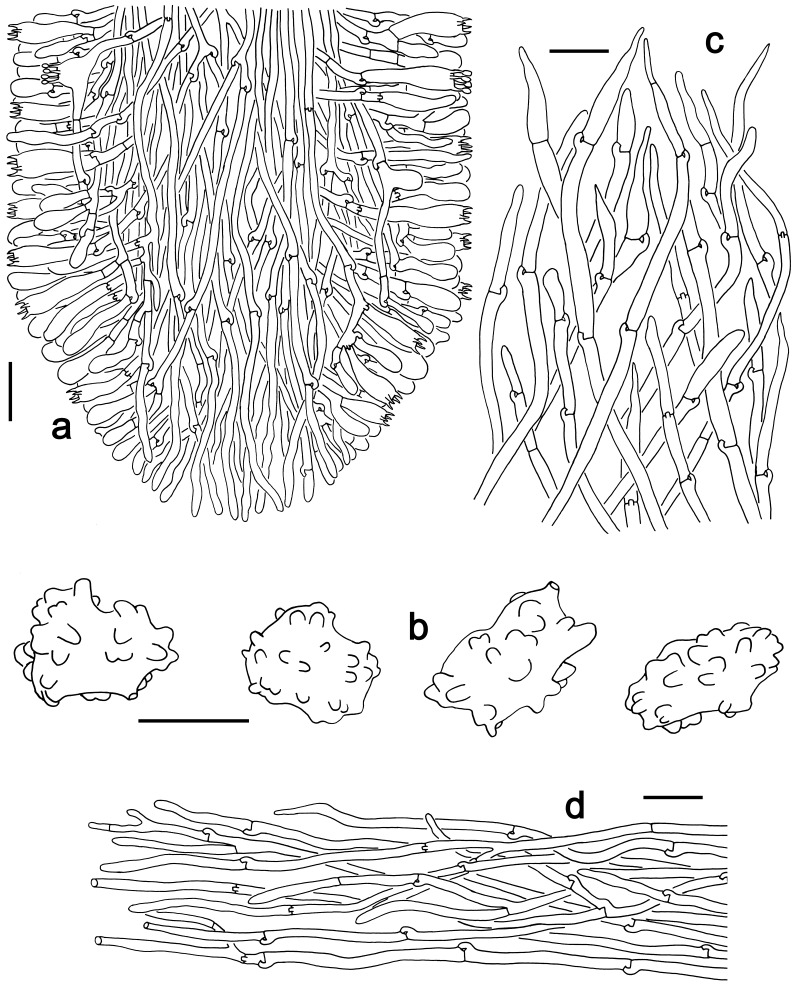
Microscopic features of *Boletopsislongipes* (KUN-HKAS 136927, holotype). **a** structure of the longitudinal section of a tube; **b** basidiospores; **c** pileipellis; **d** stipitipellis. Scale bars: a, c = 20 μm, b = 5 μm.

Hyphal system monomitic. Generative hyphae with clamp connections. Basidia thin-walled, 15–36 × 7–15 μm, clavate to cylindrical, sometimes flexuous or ventricose with a peduncle, 4-spored, clamped at base, hyaline in KOH, yellowish to pale yellow in Melzer's Reagent (Fig. 2a). Basidiospores [122/6/5] 5–7 (8) × (3.5) 4–6 μm, Q = 1–1.75, Q_m_ = 1.21 ± 0.16, globose to oblong, acyanophilic and non-dextrinoid, hyaline to pale yellow in KOH; ornamentation on the surface tuberculate and sometimes furcate (Fig. 2b; Fig. 3a, b). Cystidia absent. Tube tramal hyphae thin-walled, 2–4 μm wide, cylindrical, interwoven, hyaline in KOH. Hyphae of pileipellis thin-walled, 2–7 (9) μm wide, cylindrical, interwoven and occasionally subparallel, hyaline to faint olivaceous in KOH; terminal cells (15) 18–50 (72) × (1) 3–7.5 (9) μm (Fig. 2c). Pileal tramal hyphae thin-walled, up to 26 μm wide, inflated to filamentous, interwoven, hyaline in KOH. Hyphae of stipitipellis thin-walled, 2–7 (10) μm wide, cylindrical, interwoven or subparallel, frequently branched, faint olivaceous in KOH (Fig. 2d).

#### Diagnosis

*Boletopsislongipes* differs from other species of the genus by its pastel brown pileus when mature, relatively slender stipes 5–13 × 2–3 cm, large basidia 15–36 × 7–15 μm and its occurrence in mixed forests dominated by trees of *Quercus* sp. and *Pinusyunnanensis* at an altitude ranging from 1000 m to 2600 m.

#### Etymology

“*Longipes*” reffering to the relatively long-footed basidiomata.

#### Distribution

Currently known from Yunnan Province, China.

#### Ecology

Growing on the ground in a moist environment in mixed forests dominated by trees of the families *Fagaceae* (*Quercus* sp.) and *Pinaceae* (*Pinusyunnanensis*).

#### Notes

*Boletopsislongipes* is characterised by its black to dark grey pileus that turns pastel brown when mature, concolorous to bark brown squamules on the surface, relatively long stipes measuring 5–13 × 2–3 cm, white mycelia at the base of stipe, large basidia measuring 15–36 × 7–15 μm and solitary to gregarious occurrence in mixed forests dominated by trees of *Quercus* sp. and *Pinusyunnanensis* at an altitude ranging from 1000 m to 2600 m. This new species represents the ninth member of the genus and the third one from China. Morphologically, *B.longipes* is similar to *B.leucomelaena* and *B.watlingii* in their somewhat black to brown pileus, angular to irregular hymenophoral pores and somewhat decurrent hymenophore. However, *B.leucomelaena* is characterised by its large hymenophoral pores 1–3 per mm (vs. 2-4/mm in *B.longipes*), bright orange mycelia at the base of stipe, narrow basidia 17–27 × 5.5–8.5 μm (vs. 15–36 × 7–15 μm in *B.longipes*) and growing in dense clusters of three to ten or less often solitarily in forests dominated by trees of the genus *Picea* in Europe (Niemelä and Saarenoksa 1989, Watling and Milne 2008). *Boletopsiswatlingii* has a dark sooty-brown to greyish-brown pileus with greenish tints, white context turning greyish-violet then greyish-black (especially in the stipe) when damaged, vivid orange mycelia at the base of stipe and relatively small basidiospores measuring 4.5–4.8 (5) × 3.5–4.5 μm (Blanco-Dios 2018). *Boletopsislongipes* and *B.macrocarpa* both have large basidiomata, white context when fresh and large basidia in Yunnan Province, China. However, *B.macrocarpa* has a cream to greyish-brown pileus which is up to 21 cm wide, a stout stipe which is up to 8.5 cm long and 5 cm wide and ash-grey or concolorous with pileal surface and a distribution in pure *Pinus* forest with altitude ranging from 2400 m to 3400 m (Zhou et al. 2022).

### Boletopsis macrocarpa

Y.C. Dai, F. Wu & H.M. Zhou, 2022

D490AD58-C9A6-5919-A286-976A86DAC22D

#### Materials

- **Type status:** Other material. **Occurrence:** recordNumber: Yan-Chun Li 6792; recordedBy: Yan-Chun Li; individualID: KUN-HKAS 139404; occurrenceID: 65D47339-9FC0-5C0C-91CF-D62C94AA58C6; **Taxon:** scientificName: *Boletopsismacrocarpa*; kingdom: Fungi; phylum: Basidiomycota; class: Agaricomycetes; order: Thelephora; family: Bankeraceae; genus: Boletopsis; **Location:** higherGeography: East Asia; China; Yunnan; county: Yulong Naxi Autonomous County; locality: Laojun Mountain; verbatimElevation: 2575 m; verbatimLatitude: 27.0777°N; verbatimLongitude: 99.7100°E; **Event:** eventDate: 2024-08-29
- **Type status:** Other material. **Occurrence:** recordNumber: Zhen Wang 119; recordedBy: Zhen Wang; individualID: KUN-HKAS 120843; occurrenceID: 28E8D6D4-567F-504F-8849-420354D2F20F; **Taxon:** scientificName: *Boletopsismacrocarpa*; kingdom: Fungi; phylum: Basidiomycota; class: Agaricomycetes; order: Thelephora; family: Bankeraceae; genus: Boletopsis; **Location:** higherGeography: East Asia; China; Yunnan; county: Diqing Tibetan Autonomous Prefecture; locality: Shangri-La, Geza Village; verbatimElevation: 3377 m; verbatimLatitude: 28.0173°N; verbatimLongitude: 99.7883°E; **Event:** eventDate: 2019-08-16
- **Type status:** Other material. **Occurrence:** recordNumber: Xiang-Hua Wang 6104; recordedBy: Xiang-Hua Wang; individualID: KUN-HKAS 116743; occurrenceID: BD67534C-6120-5E0D-B08A-4E81AB7328D5; **Taxon:** scientificName: *Boletopsismacrocarpa*; kingdom: Fungi; phylum: Basidiomycota; class: Agaricomycetes; order: Thelephora; family: Bankeraceae; genus: Boletopsis; **Location:** higherGeography: East Asia; China; Yunnan; county: Diqing Tibetan Autonomous Prefecture; locality: Shangri-La, Geza Village; verbatimElevation: 3377 m; verbatimLatitude: 28.0173°N; verbatimLongitude: 99.7883°E; **Event:** eventDate: 2019-08-16

#### Description

Basidiomata annual, medium to large, centrally stipitate, solitary to gregarious (Fig. 1d, e). Pileus 6.5–21 cm in diameter, roundish to irregular, slightly depressed at centre, cream (5A2) to greyish-brown (5E6), becoming pale brownish-grey (5D3) to black when dry; margin occasionally cream, undulate. Context white (1A1), becoming pale mouse-grey (2B/C2) when dry. Hymenophore slightly decurrent; surface white (1A1) to cream (5A2), becoming clay buff (6D4) to fawn (7D/E4) when dry, pores subangular to angular or irregular, 1–3 per mm; tubes concolorous with hymenophoral surface, turning fawn (6D7) when bruised, up to 0.5 cm long. Stipe up to 8.5 cm long and 5.5 cm wide, solid, subcylindrical, pale ash-grey (17B1) or concolorous with pileal surface.

**Figure 4.**
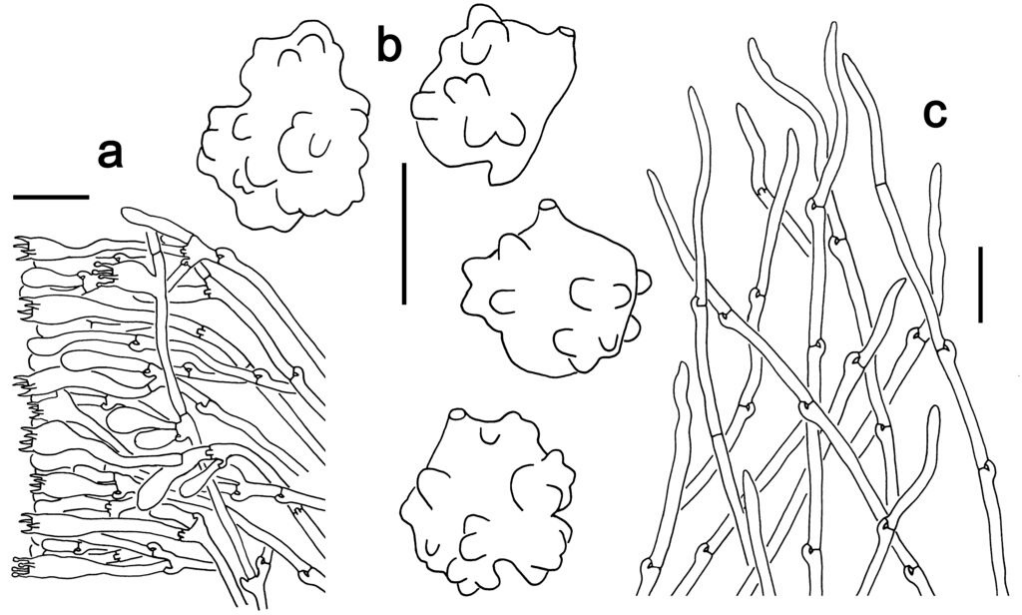
Microscopic features of *Boletopsismacrocarpa* (KUN-HKAS 120843). **a** basidia and basidioles; **b** basidiospores; **c** pileipellis. Scale bars: a,c = 20 μm, b = 5 μm.

Hyphal system monomitic. Generative hyphae with clamp connections. Basidia thin-walled, 18–45 × 5.5–10 μm, clavate to cylindrical, sometimes flexuous or ventricose with a peduncle, 4-spored, clamped at base, hyaline in KOH (Fig. 4a). Basidiospores [60/3/3] (4.5) 5–6 × (3.5) 4–5 (5.5) μm, Q = 1–1.38 (1.5), Q_m_ = 1.20 ± 0.13, globose to oblong, acyanophilic and non-dextrinoid, hyaline to pale yellow in KOH; ornamentation on the surface tuberculate and sometimes furcate (Fig. 3c, d; Fig. 4b). Cystidia absent. Tube tramal hyphae thin-walled, 2–4 μm wide, cylindrical, interwoven and occasionally subparallel in a bunch, hyaline in KOH. Hyphae of pileipellis thin-walled, 3–9 μm wide, cylindrical, interwoven and occasionally subparallel, hyaline to faint olivaceous in KOH; terminal cells 22–53 (56) × (3) 4–7 (9) μm (Fig. 4c). Pileal tramal hyphae thin-walled, 4–44 μm wide, inflated, sometimes branched, interwoven, hyaline in KOH. Hyphae of stipitipellis thin-walled, 3–20 μm wide, cylindrical, parallel, rarely branched, faint olivaceous in KOH.

#### Distribution

Currently known from Yunnan Province, China.

#### Ecology

Growing on the ground in the pure coniferous forests dominated by trees of the genus *Pinus* (*Pinusyunnanensis*) at an altitude ranging from 2400 m to 3400 m.

#### Notes

*Boletopsismacrocarpa* is characterised by its large pileus (6.5–21 cm wide) with cream to greyish-brown surface, white to cream hymenophoral surface, solitary to gregarious in coniferous forests of *Pinusyunnanensis* in slightly dry environments at a high altitude ranging from 2400 m to 3400 m. Morphologically, *B.macrocarpa* is similar to *B.grisea* in their somewhat greyish-tinged pileus, white to cream hymenophoral surface and distribution in the pure *Pinus* forests. However, *B.grisea* originally described from Europe is characterised by its grey pileus, short stipes 2–6 cm long (vs. 8.5 cm long in *B.macrocarpa*), small pores 2–4/mm (vs. 1–3/mm in *B.macrocarpa*), gloeoplerous hyphae in pileipellis and narrow basidiospores (4.2) 5–6 (6.5) × (3.2) 3.9–4.5 (5.1) [vs. (4.5) 5–6 × (3.5) 4–5 (5.5) μm in *B.macrocarpa*] (Peck 1873, Niemelä and Saarenoksa 1989). In our re-examination of *B.macrocarpa*, we found larger basidia measuring 18–45 × 5.5–10 μm (14–19 × 6–7 μm in the protologue) and wider hyphae of pileal trama measuring 4–44 μm wide (5–25 μm wide in original description) than those in the protologue of Zhou et al. (2022).

### Boletopsis tibetana

Y.C. Dai, F. Wu & H.M. Zhou, 2022

6B062519-C669-5EEA-A3A9-E36FDCE0B34D

#### Materials

- **Type status:** Other material. **Occurrence:** recordNumber: Bang Feng 1680; recordedBy: Bang Feng; individualID: KUN-HKAS 94064; occurrenceID: B7750492-4BE1-55F6-9AD7-B61D18FA7558; **Taxon:** scientificName: *Boletopsistibetana*; kingdom: Fungi; phylum: Basidiomycota; class: Agaricomycetes; order: Thelephorales; family: Bankeraceae; genus: Boletopsis; **Location:** higherGeographyID: East Asia; China; Tibet; county: Linzhi; locality: Lulang Town, Bayi, Zhaxigang Village; verbatimElevation: 3350 m; **Event:** eventDate: 2014-08-01

#### Description

Basidiomata annual, medium, centrally stipitate, solitary to gregarious (Fig. 1f). Pileus up to 12 cm in diameter, convex or irregular, clay buff (5C3) to dark brown (5F7), becoming greyish-brown (4C3) to black when dry; margin undulate and incurved. Context white (1A1), becoming pale brown (1B3) when dry. Hymenophore slightly decurrent, surface white (1A1), becoming clay buff (6D4) to fawn (7D/E4) when dry; hymenophoral pores round to angular or irregular, 1–4 per mm; tubes concolorous with hymenophoral surface, darkening when bruised. Stipe up to 9 cm long and 2 cm wide, solid, subcylindrical or tapering towards the base, concolorous with pileal surface.

**Figure 5.**
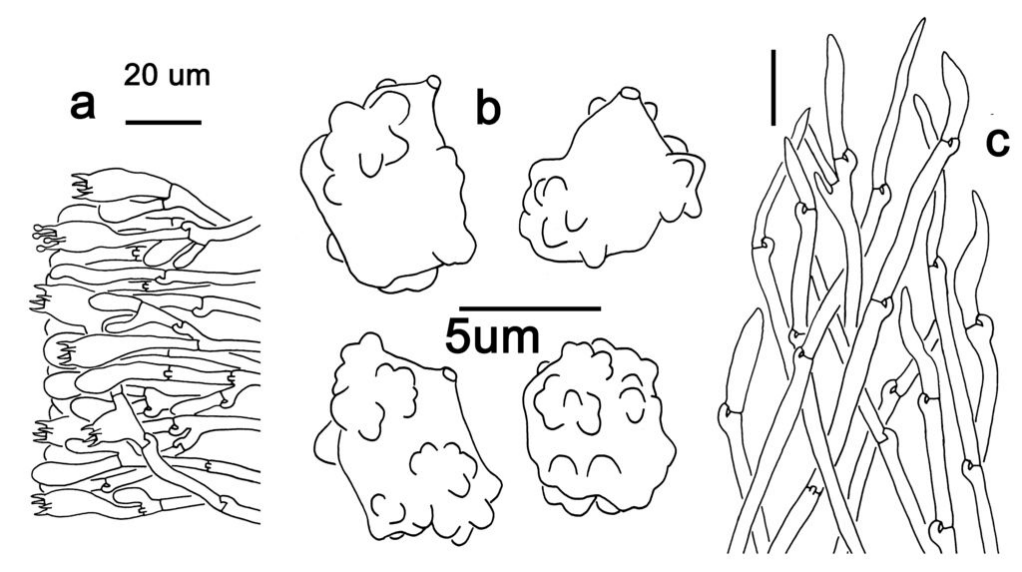
Microscopic features of *Boletopsistibetana* (KUN-HKAS 94064). **a** basidia and basidioles; **b** basidiospores; **c** pileipellis. Scale bars: a, c = 20 μm, b = 5 μm.

Hyphal system monomitic. Generative hyphae with clamp connections. Basidia thin-walled, 19–25 (35) × 8–11 μm, clavate to cylindrical, sometimes flexuous or ventricose with a peduncle, 4-spored, clamped at base, hyaline in KOH (Fig. 5a). Basidiospores [20/1/1] 5–6 (6.5) × 4–5.5 (6) μm, Q = 1–1.25 (1.5), Q_m_ = 1.12 ± 0.13, globose to oblong, acyanophilic and non-dextrinoid, hyaline to pale yellow in KOH; ornamentation on the surface tuberculate and sometimes furcate (Fig. 3e, f; Fig. 5b). Cystidia absent. Tube tramal hyphae thin-walled, 2–4 μm wide, cylindrical, sometimes branched, interwoven and occasionally subparallel in a bunch, hyaline in KOH. Hyphae of pileipellis thin-walled, 4–10 μm wide, cylindrical with finger-shaped tips, sometimes branched, interwoven, faint olivaceous in KOH; terminal cells (37) 47–77 × 4–6 μm (Fig. 5c). Pileal tramal hyphae thin-walled, 6–30 μm wide, inflated, rarely branched, interwoven and occasionally subparallel in a bunch, hyaline in KOH. Hyphae of stipitipellis thin-walled to slightly-thick wall (up to 0.7 μm thick), 2–8 μm wide, cylindrical, interwoven, frequently branched, hyaline in KOH.

#### Distribution

Currently known from Tibet, China.

#### Ecology

Growing on the ground in forests dominated by trees of the families *Fagaceae* (*Quercus* sp.) and *Pinaceae* (*Piceabalfouriana*) or in pure *Picea* forests dominated by *P.balfouriana* at an altitude ranging from 2900 m to 3350 m.

#### Notes

*Boletopsistibetana* is characterised by its clay buff to dark brown pileus, gloeoplerous hyphae in pileipellis and context, solitary to gregarious in mixed forests dominated by *Picea* and *Quercus* or in pure *Picea* forests at an altitude ranging from 2900 m to 3350 m. *Boletopsistibetana* resembles *B.leucomelaena* by its solitary to gregarious habitat, frequently branched hyphae of stipitipellis and distribution in forests dominated by tres of the genus *Picea*. However, *B.leucomelaena* has greyish sepia or black-brown pileus often with a tinge of magenta, large pores (1–3 per mm) and a distribution in Europe (Niemelä and Saarenoksa 1989). In our re-examination of *B.tibetana*, we found wider basidia (19–35 × 8–11 μm) than those in the protologue (13–25 × 6–8 μm) (Zhou et al. 2022).

## Identification Keys

### Key to the accepted species of Boletopsis worldwide

**Table:** 

1	Species distributed in Southern Hemisphere, associated with *Nothofagus* trees	*B.nothofagi*
–	Species distributed in Northern Hemisphere, associated with trees of the genera *Picea* or *Pinus*	2
2	Species distributed in East Asia	3
–	Species distributed in North Africa, Europe and North America	5
3	Pileus cream to greyish-brown; stipe stout, up to 8.5 cm long and 5 cm wide; distributed in pure *Pinus* forest; currently known from Yunnan Province	*B.macrocarpa*
–	Pileus clay buff to dark brown or black to dark grey then pastel brown, without cream tinge; stipe relatively slender, not more than 3 cm wide; distributed in pure *Picea* forests or in mixed forests dominated by trees of the genera *Picea* and *Quercus* or in mixed forests dominated by trees of the genera *Pinus* and *Quercus*; currently known from Yunnan Province and Tibet	4
4	Pileus medium-sized, up to 12 cm wide; stipe up to 9 cm long; distributed in mixed forests dominated by trees of the genera *Picea* and *Quercus* or in pure *Picea* forests, with an altitude ranging from 2900 m to 3350 m; currently known from Tibet	*B.tibetana*
–	Pileus large, up to 17 cm wide; stipe up to 13 cm long; distributed in mixed forests dominated by trees of the genera *Pinus* and *Quercus*, with an altitude ranging from 1000 m to 2600 m; currently known from Yunnan Province	*B.longipes*
5	Basidiospores < 5 μm long	*B.watlingii*
–	Basidiospores ≥ 5 μm long	6
6	Pileus dull orange when fresh	*B.smithii*
–	Pileus grey or brown to black when fresh without orange tinge	7
7	Pileus medium-sized, up to 10 cm wide, greyish sepia to black-brown; usually associated with trees of the genus *Picea*	*B.leucomelaena*
–	Pileus large, up to 20 cm wide; usually associated with trees of the genus *Pinus*	8
8	Pileus grey to ochraceous brown or dark brown; context pale grey to grey, becoming pale red when cut, turning green in KOH; predominantly distributed in Mediterranean regions	*B.mediterraneensis*
–	Pilelus grey-white to grey-brown without ochraceous brown tinge; context white, becoming lilac-grey when cut, greenish before turning black in KOH; predominantly distributed in Eurosiberian Region	*B.grisea*

## Analysis

**Figure 6.**
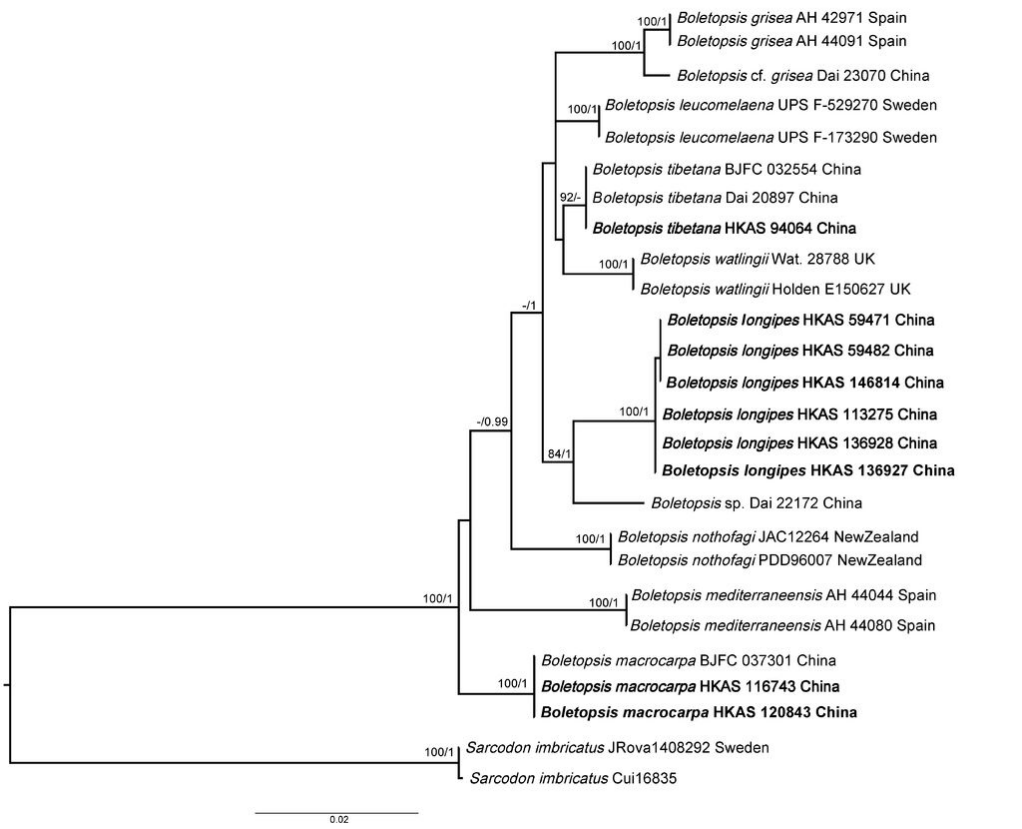
Phylogenetic relationships of *Boletopsis* species, based on the combined dataset (ITS + nrLSU) using Maximum Likelihood and Bayesian Inference approaches (only the ML topology is shown). Bootstrap frequencies ≥ 75% and posterior probabilities ≥ 0.95 are shown above the branches. Sequences newly generated in this study are in bold.

In our phylogenetic analyses, 47 sequences including seven of the eight accepted species and the new species of *Boletopsis* were used. The combined dataset (ITS + nrLSU) is 2156 bp in length. Due to the similarity of phylogenetic topologies from ML and BI analyses, only the ML tree is shown (Fig. 6). Based on our phylogenetic analyses, eight *Boletopsis* species were recognised including two known species, viz. *B.macrocarpa* and *B.tibetana*, one new species *B.longipes* described in this study and two potential new species which are recognised by Zhou et al. (2022) and this study, but unclarified due to the paucity of materials (samples: Dai 23070 labelled as Boletopsiscf.grisea and Dai 22172 labelled as *Boletopsis* sp., respectively). Sequences of six collections (HKAS 59471, HKAS 59482, HKAS 113275, HKAS 136927, HKAS 136928 and HKAS 146814) of the new species *B.longipes* formed an independent lineage and clustered together with *Boletopsis* sp. (sample: Dai 22172) with high support values (BS = 84%; PP = 1).

## Discussion

In this study, three *Boletopsis* species from China were illustrated and documented. There are also two collections Dai 23070 and Dai 22172 from China (Zhou et al. 2022) representing two potential new species that can not be clarified due to the paucity of materials and further collections and analyses are required to clarify their taxonomic status. The presence of cystidia in *Boletopsis* is only found in *B.mediterraneensis* (Vizzini et al. 2023) and “cystidia-like elements” appear in *B.nothofagi* (Cooper and Leonard 2012). However, similar elements are regarded as basidioles in *B.tibetana* (Zhou et al. 2022). In addition, the cystidia described in *B.mediterraneensis* sometimes can be interpreted either as basidioles or cystidia (Vizzini et al. 2023).

## Supplementary Material

XML Treatment for Boletopsis longipes

XML Treatment for Boletopsis macrocarpa

XML Treatment for Boletopsis tibetana

## Figures and Tables

**Figure 1a.**
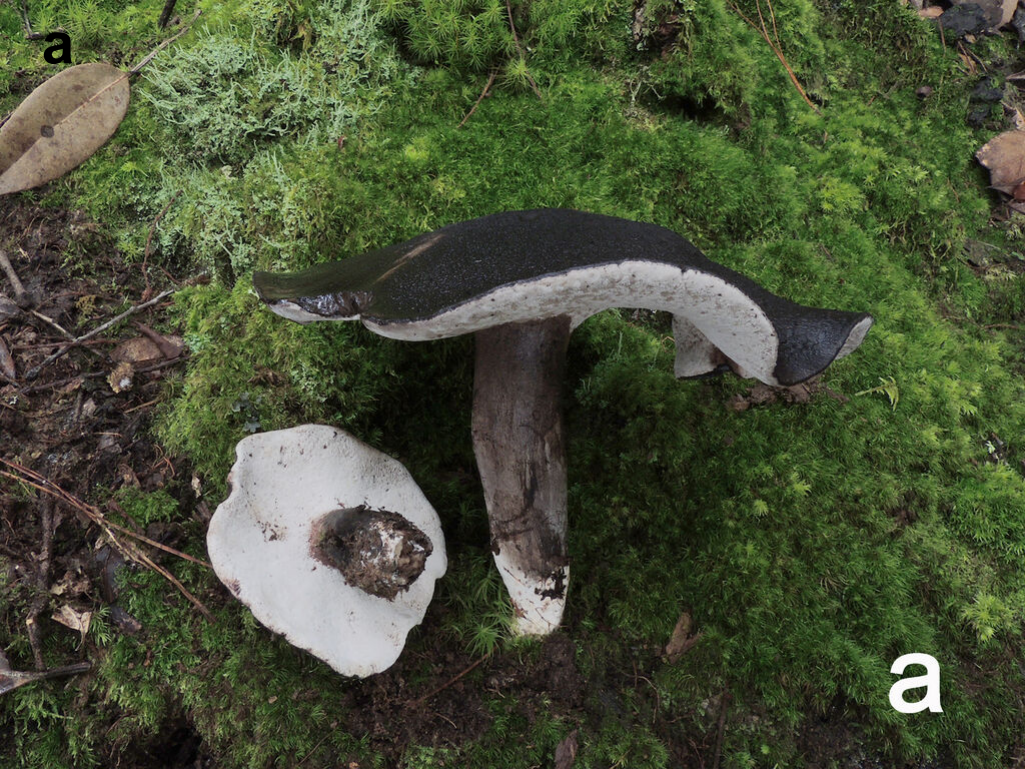
*Boletopsislongipes* (KUN-HKAS 136927, holotype, photo by Yan-Chun Li);

**Figure 1b.**
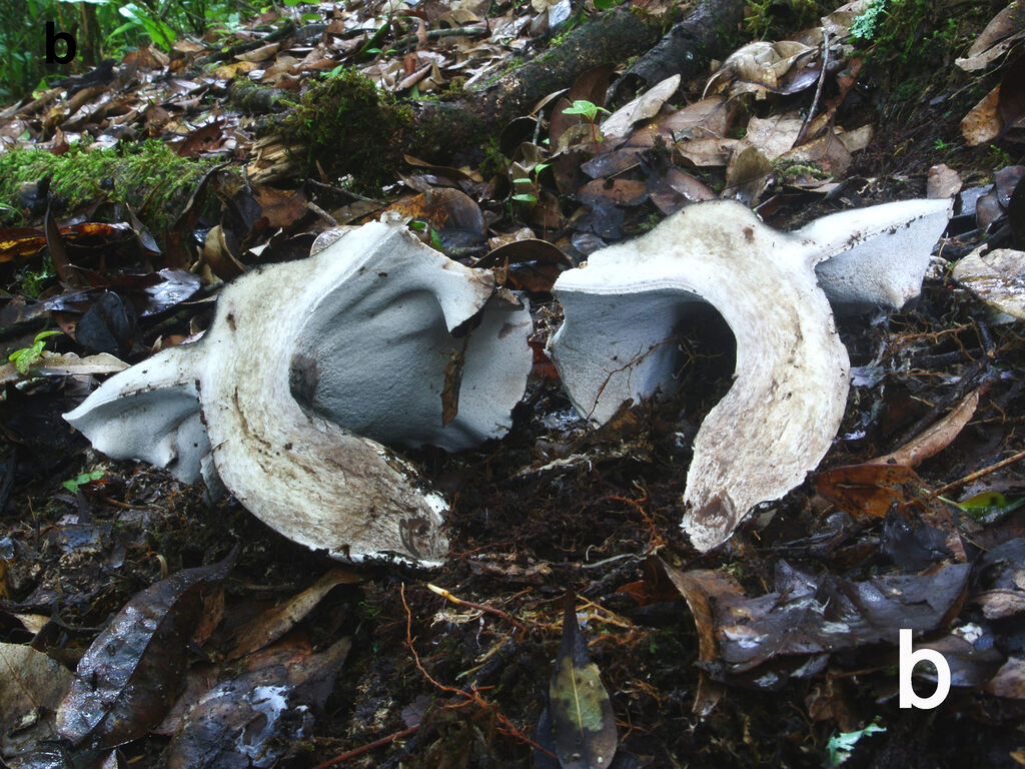
*Boletopsislongipes* (KUN-HKAS 113275, photo by Mei-Xiang Li);

**Figure 1c.**
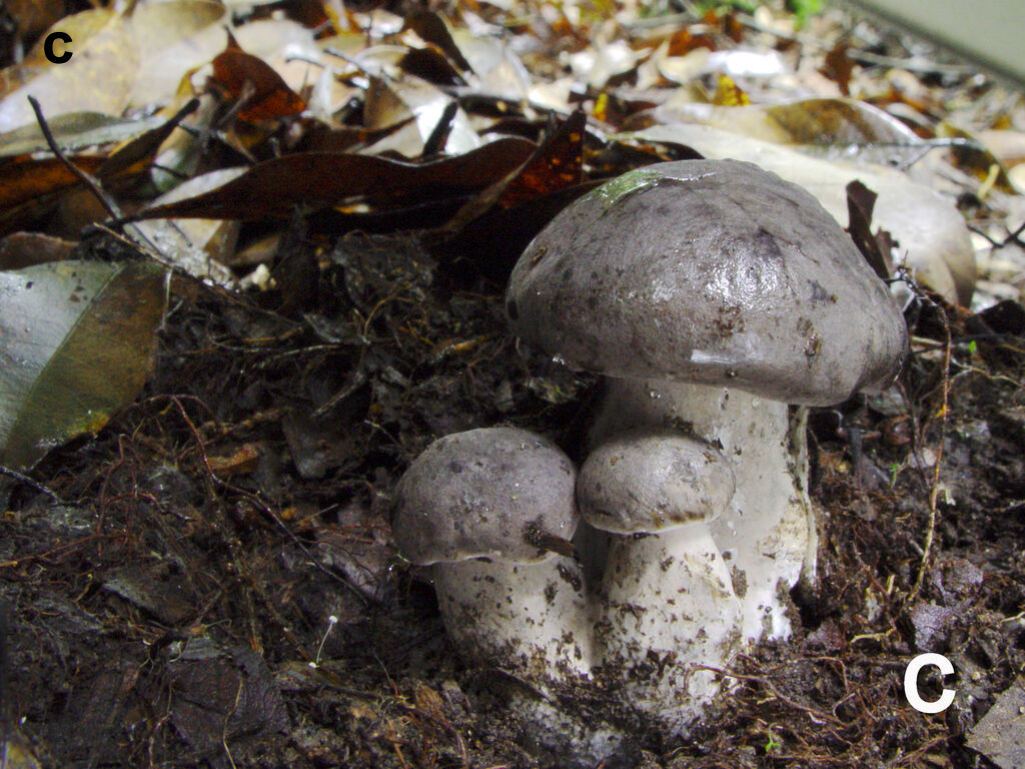
*Boletopsislongipes* (KUN-HKAS 59482, photo by Yan-Chun Li);

**Figure 1d.**
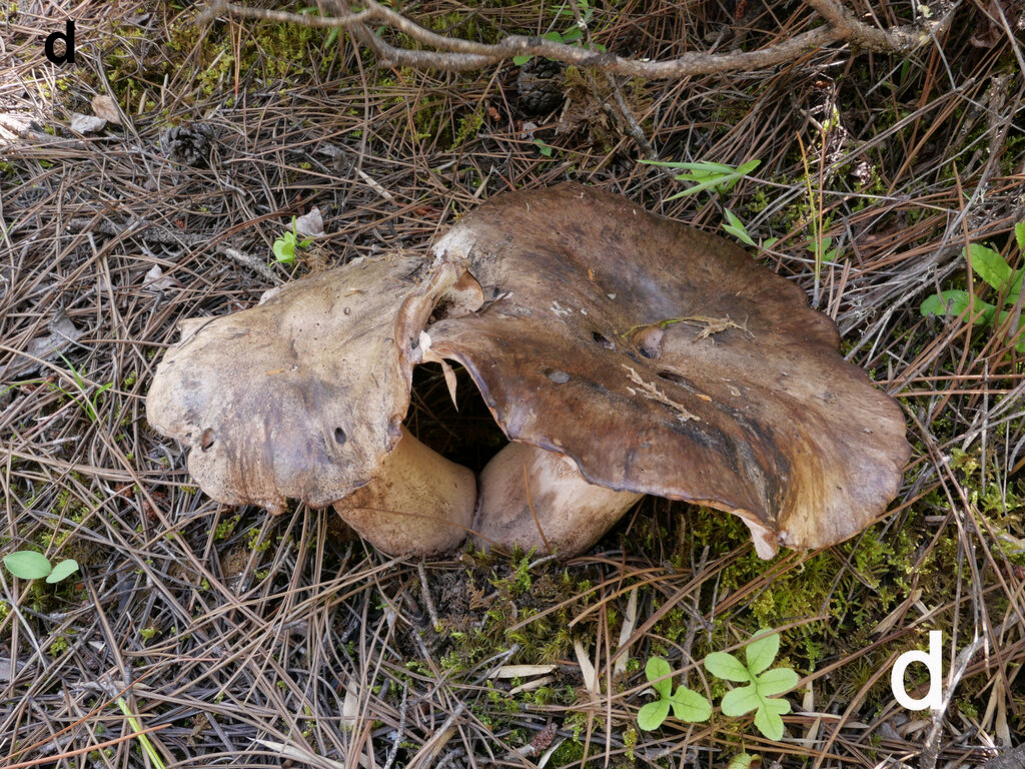
*Boletopsismacrocarpa* (KUN-HKAS 120843, photo by Zhen Wang);

**Figure 1e.**
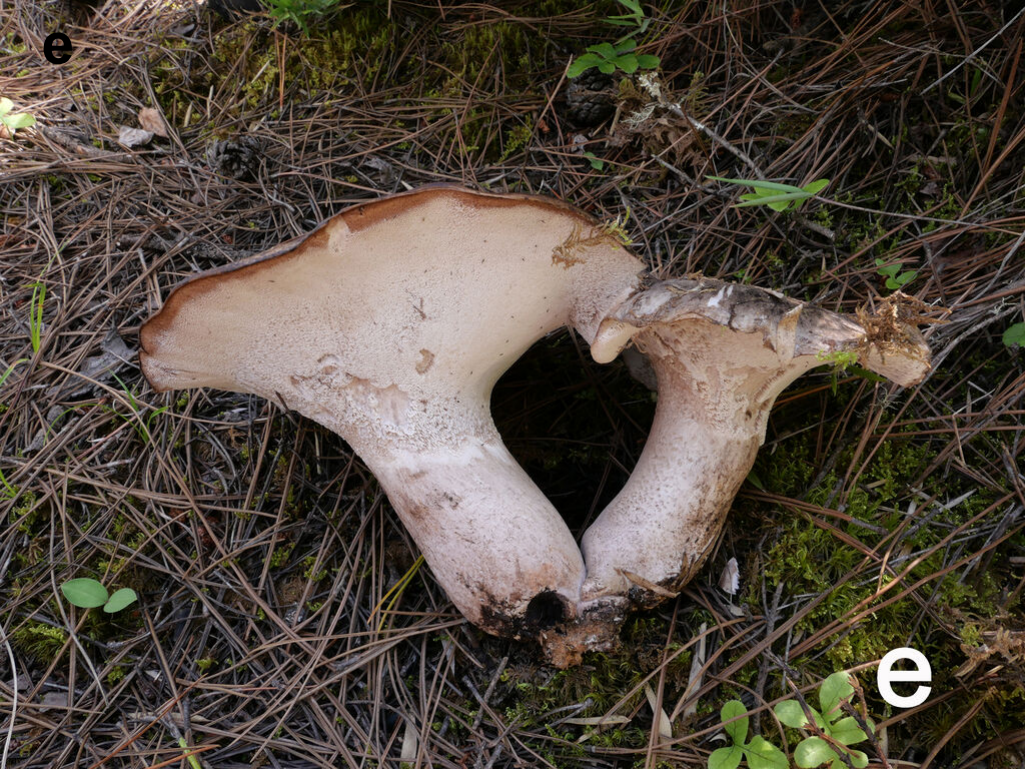
*Boletopsismacrocarpa* (KUN-HKAS 120843, photo by Zhen Wang);

**Figure 1f.**
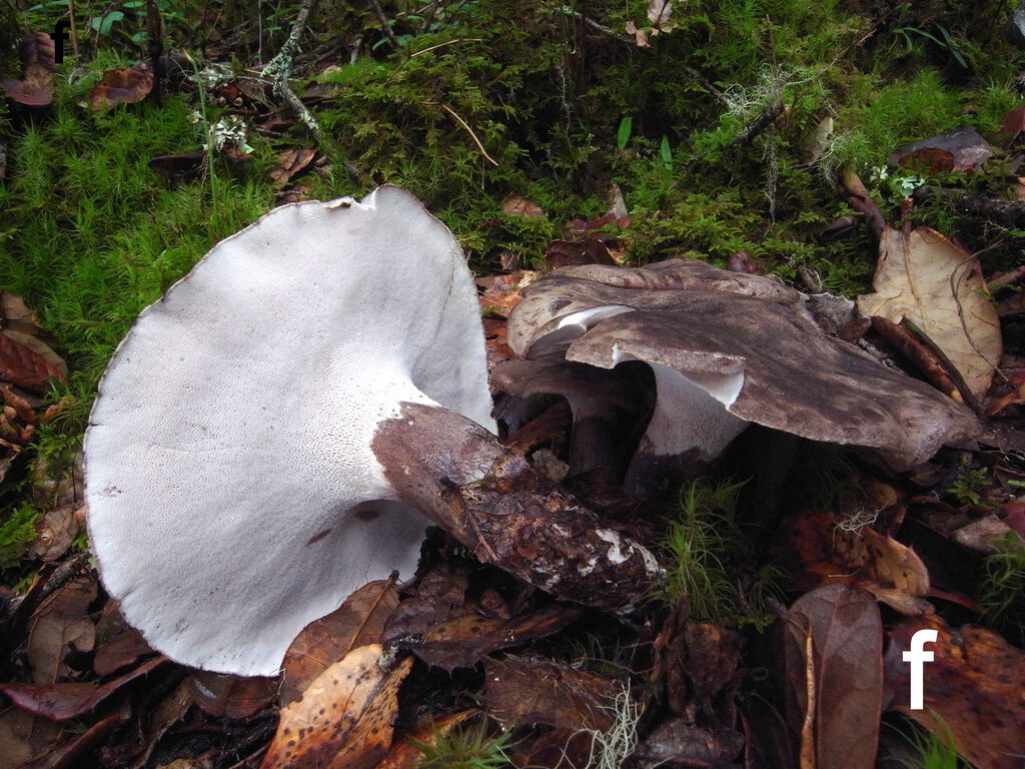
*Boletopsistibetana* (KUN-HKAS 94064, photo by Bang Feng).

**Figure 3a.**
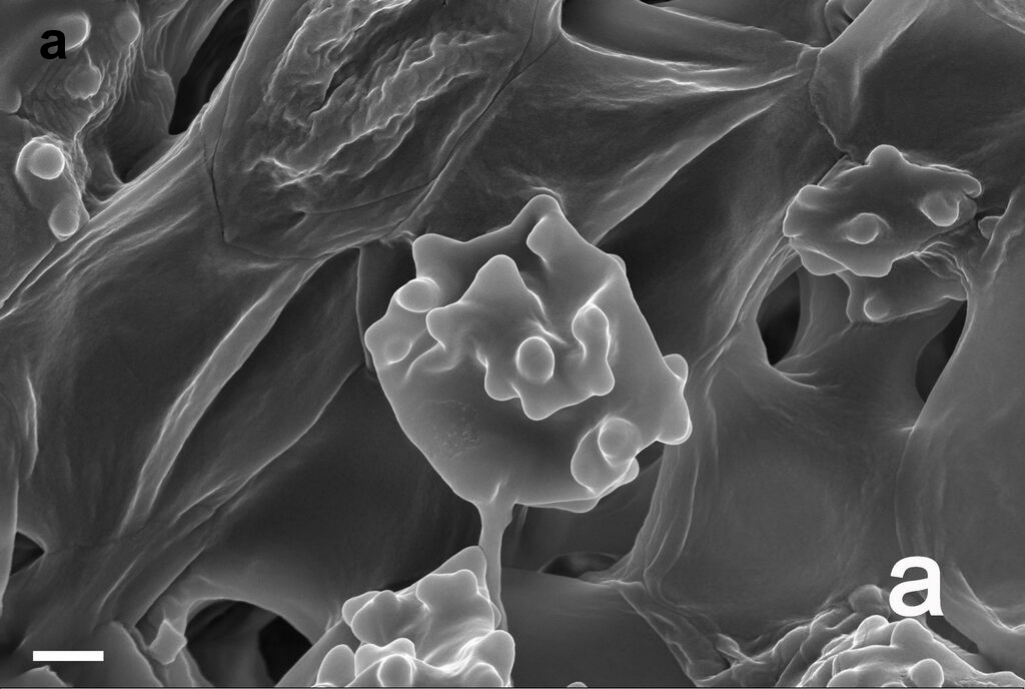
*Boletopsislongipes* (KUN-HKAS 136927, type);

**Figure 3b.**
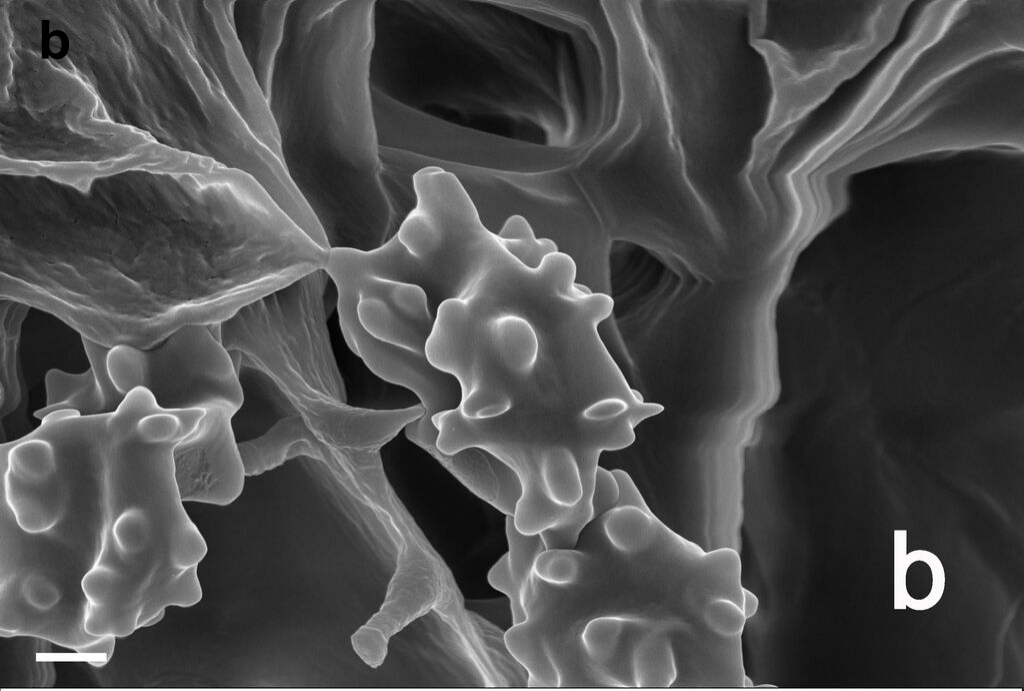
*Boletopsislongipes* (KUN-HKAS 136927, type);

**Figure 3c.**
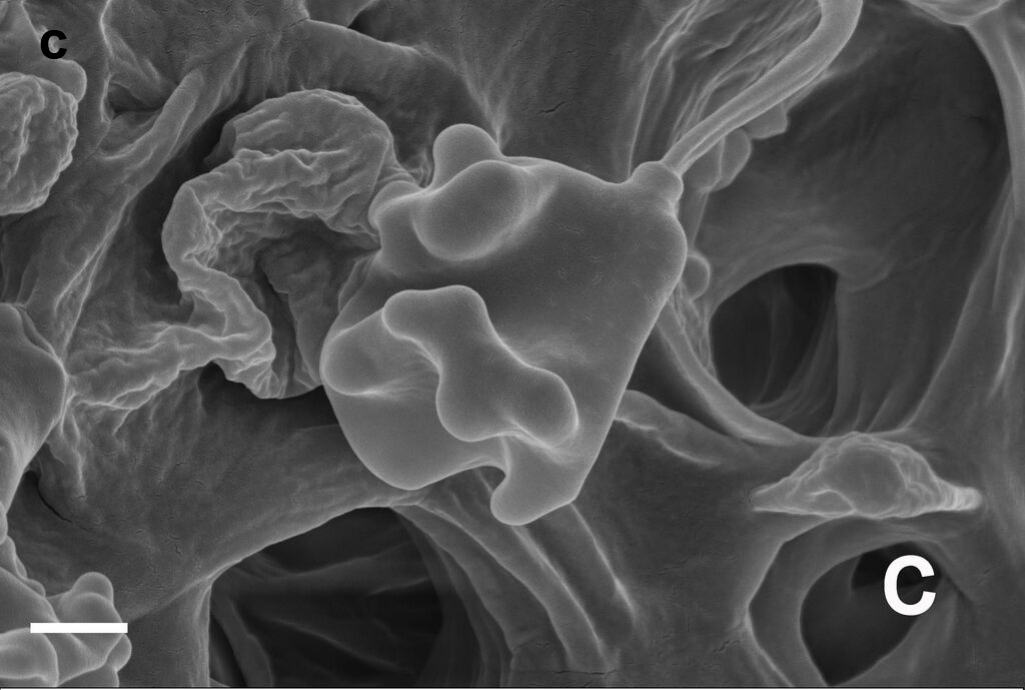
*Boletopsismacrocarrpa* (KUN-HKAS 116743);

**Figure 3d.**
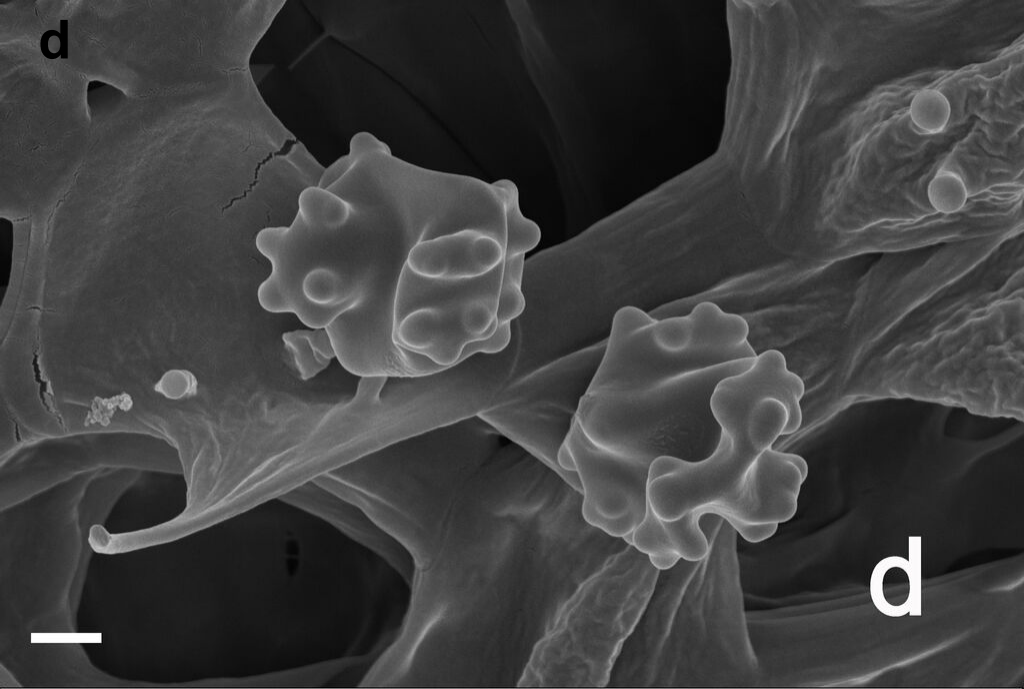
*Boletopsismacrocarrpa* (KUN-HKAS 116743);

**Figure 3e.**
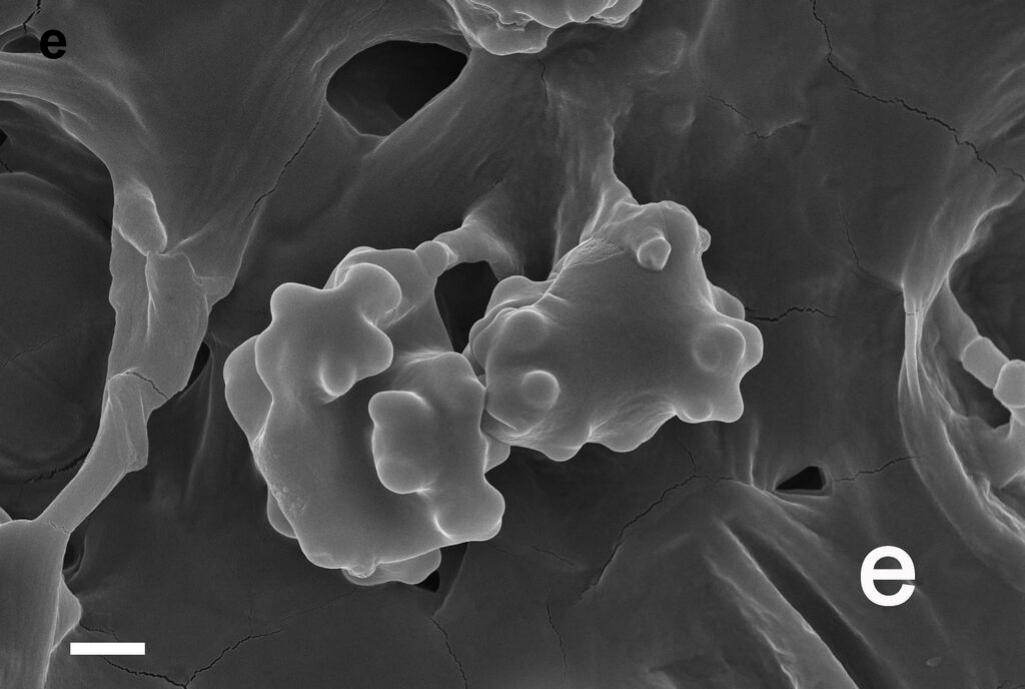
*Boletopsistibetana* (KUN-HKAS 94064);

**Figure 3f.**
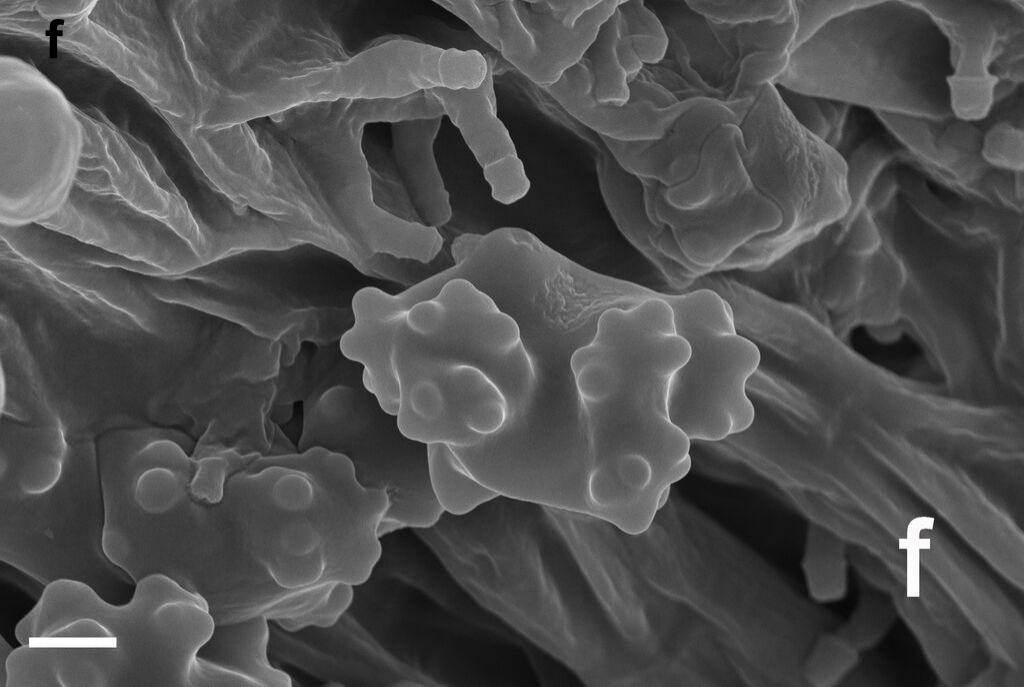
*Boletopsistibetana* (KUN-HKAS 94064).

**Table 1.** Taxa, vouchers, countries and GenBank accession numbers of the species used in this study. Sequences obtained in this study were shown in bold.

Species	Voucher	Country	ITS	nrLSU	Reference
*Boletopsisgrisea*	AH 42971	Spain	MN536747	MN535642	Crous et al. (2019)
*Boletopsisgrisea*	AH 44091	Spain	MN536748	MN535643	Crous et al. 2019
Boletopsiscf.grisea	Dai 23070	China	OL673003	OL672990	Zhou et al. 2022
*Boletopsisleucomelaena*	UPS F-529270	Sweden	MN536741	MN535640	Crous et al. 2019
*Boletopsisleucomelaena*	UPS F-173290	Sweden	MN536739	MN535638	Crous et al. 2019
***Boletopsislongipes***	**HKAS 59471**	**China**	**PQ182880**	**PQ182688**	**This study**
***Boletopsislongipes***	**HKAS 59482**	**China**	**PQ182881**	**PQ182689**	**This study**
***Boletopsislongipes***	**HKAS 113275**	**China**	**PQ182879**	**PQ182690**	**This study**
***Boletopsislongipes***	**HKAS 136927**	**China**	**PQ182877**	**PQ182691**	**This study**
***Boletopsislongipes***	**HKAS 136928**	**China**	**PQ182878**	**PQ182692**	**This study**
***Boletopsislongipes***	**HKAS 146814**	**China**	**PV546625**	**PV544815**	**This study**
*Boletopsismacrocarpa*	BJFC 037301	China	OL673005	OL672992	Zhou et al. 2022
***Boletopsismacrocarpa***	**HKAS 116743**	**China**	**PQ182882**	**PQ182693**	**This study**
***Boletopsismacrocarpa***	**HKAS 120843**	**China**	**PQ182883**	-	**This study**
*Boletopsismediterraneensis*	AH 44080	Spain	MN536723	MN535629	Crous et al. 2019
*Boletopsismediterraneensis*	AH 44044	Spain	MN536715	MN535625	Crous et al. 2019
*Boletopsisnothofagi*	PDD 96007	New Zealand	JQ417193	MW683928	Cooper and Leonard 2012
*Boletopsisnothofagi*	JAC 12264	New Zealand	-	MW683890	Cooper and Leonard 2012
*Boletopsis* sp.	Dai 22172	China	OL673011	OL672998	Zhou et al. 2022
*Boletopsistibetana*	BJFC 032554	China	OL673013	OL673000	Zhou et al. 2022
*Boletopsistibetana*	Dai 20897	China	OL673012	OL672999	Zhou et al. 2022
***Boletopsistibetana***	**HKAS 94064**	**China**	**PQ182884**	-	**This study**
*Boletopsiswatlingii*	Wat. 28788	United Kingdom	DQ408767	-	Watling and Milne 2006
*Boletopsiswatlingii*	Holden E150627	United Kingdom	DQ408766	-	Watling and Milne 2006
*Sarcodonimbricatus*	Cui 16835	-	OR761670	OR761791	GenBank
*Sarcodonimbricatus*	JRova 1408292	Sweden	MK602746	MK602746	Larsson et al. 2019
